# An Integrated Music and Artificial Intelligence System in Support of Pediatric Neurorehabilitation

**DOI:** 10.3390/healthcare10102014

**Published:** 2022-10-12

**Authors:** Federico Baldisseri, Arturo Maiani, Edoardo Montecchiani, Francesco Delli Priscoli, Alessandro Giuseppi, Danilo Menegatti, Vincenzo Fogliati

**Affiliations:** 1 Department of Computer, Control and Management Engineering (DIAG), University of Rome “La Sapienza”, Via Ariosto 25, 00185 Rome, Italy; 2 CRAT, Via Giovanni Nicotera 29, 00195 Rome, Italy

**Keywords:** artificial intelligence, music, neural network classification

## Abstract

This study aims at the implementation of an Artificial Intelligence approach to the use of music for supporting the neurorehabilitation of children with brain injuries or neurological difficulties.The output of this study will be the implementation of an app for mobile devices with games to be played by pediatric patients, allowing time for their cognitive and motor abilities to recover while enjoying pleasant activities. In particular, a Neural Network Classification approach is proposed in order to automatically adapt the game difficulty to the current cognitive capabilities of the child.

## 1. Introduction

Music, as a powerful and versatile stimulus for the brain, has been used in neurorehabilitation as a promising complementary strategy in combination with other therapies for individuals with neurological disorders [1]. The use of music in a rehabilitation framework has been studied and applied in scientific approach since the 1950s, and is an area currently experiencing growth. Moreover, cognitive rehabilitation via the use of digital tools to increase cognitive performance through exercises and games is spreading in experimental clinical settings. It has been proven that this type of intervention improves cognitive functions such as working memory, attention and processing speed [2].

This study aims at implementing a selection of the music rehabilitation techniques present in the academic literature with the support of AI.

The main contributions of this work are:

- A review of the music rehabilitation techniques in the academic literature: in particular, five of them are selected.
- A review of the academic literature on AI methods for autonomous game difficulty adaptation.
- The design of five rehabilitative games based on the selected music rehabilitation techniques, in which the difficulty is automatically adapted via a proposed Neural Network Classification architecture.

The objective is to improve the conditions afflicting children not only by enhancing cognitive abilities, but also by exploiting the emotional impact of music, by unleashing children’s creativity, and by encouraging collaborative interactions between children.

The rest of the paper is organized as follows: Section 2 introduces the selected music rehabilitation techniques; Section 3 presents the reviewed AI methods for autonomous game difficulty adaptation; Section 4 introduces the five rehabilitative games; Section 5 discusses the proposed Neural Network Classification algorithm; Section 6 provides the conclusions and suggests future works.

## 2. Music Rehabilitation Techniques

This section reports the outcome of a study reported in the literature on music rehabilitation techniques.

In recent years, the techniques for morphological visualization of brain activity have shown that music activates several brain structures involved in cognitive, motor and emotional processing, such sensory motor areas, premotor cortex, basal ganglia and cerebellum [3]. It is clear that musical stimuli are able to induce neurophysiological modifications, both in the short and long term, which convey modulations inside neuronal networks [4].

Moreover, the emotional content of music can also be used to modulate the tone of mood, which is often a co-morbidity in neurodegenerative disorders. Indeed, musical elements can bring into play important aesthetic, psychological and motivational dimensions, from which significant changes can be derived in the approach with the patient and in the rehabilitative action. Music, by provoking the secretion of neurohormones such as serotonin and dopamine, is experienced as a joyous and rewarding activity through activity changes in the amygdala and ventral striatum [5].

Additionally, the inter-subjective component originating from specific music rehabilitation techniques such as improvisation, can activate brain areas involved not only in motor activation and regulation, but also in emotional and behavioural processing [6].

Thus, music rehabilitation techniques extend beyond performance, exerting a significant potential action on neurological diseases and providing adequate relational support for psychological problems that are often associated with such diseases. The process of making music renders neurorehabilitation more enjoyable and can remediate impaired neural processes or neural connections by engaging and linking brain regions that might otherwise not be linked together. Lastly, the music rehabilitation techniques also constitute a cost-effective and easily applicable rehabilitation strategy, both independent of traditional methods and complementary to them.

### 2.1. Rhythmic Auditory Stimulation

Rhythmic auditory stimulation (RAS) is a music rehabilitation technique used for supporting the rehabilitation of movements that are naturally rhythmic, such as gait. It consists of presenting a series of auditory stimuli at a fixed cadence to which the patient’s movements are to be synchronized.

RAS has proved very helpful in the neurorehabilitation of paresis [7]: an impairment of voluntary movement, which weakens functional capabilities of the Basal Ganglia. The primary pathway of Basal Ganglia is the Supplementary Motor Area, which is involved in the initiation of internally generated movements. Stroke patients generally have difficulties with voluntary movement. However, there is another pathway capable of performing movements: the Premotor Area. The Premotor Area is involved in movement in response to external cues. In fact, research has shown that external cues help stroke patients to move better. This is because RAS employs this alternative pathway that is less affected by the disease. Indeed, music can be used to target this pathway and thus help enhance motor rehabilitation.

### 2.2. Melodic Intonation Therapy

Melodic Intonation Therapy (MIT) is a singing-based music rehabilitation technique for supporting the rehabilitation of individuals with speech impairments. It has been observed that patients with speech impairments may be able to sing lyrics better than they can speak the same words [8]. Therefore, MIT was developed in order to engage sensorimotor networks associated with articulation in the unaffected brain areas.

MIT can be applied alternatively by intonating words and simple phrases using a melodic contour which follows the prosody of speech, or by rhythmic tapping of the hand with each syllable production as a catalyst for fluency.

MIT has proven to be useful in helping non-fluent aphasic patients to recover the ability to pronounce words and sentences [5].

In fact, musical activity has proven to be a powerful stimulus for brain plasticity, a concept that describes the ability of neuronal networks to adapt through growth and reorganization [9]. Functional restoration relies upon the ability of spared neurons to compensate for lost functions by growing neurites and forming new synapses to rebuild and remodel the injured networks [10]. This functional restoration can be achieved both by targeted training of the weakened function and by increasing the overall brain activity.

### 2.3. Musical Mnemonics Training

Musical Mnemonics Training (MMT) is a music rehabilitation technique used for enhancing memory recovery and improvement. In MMT, music is employed as a mnemonic device to sequence and organize information and add meaning, pleasure, emotion, and motivation in order to enhance the person’s ability to learn and recall the information involved. The MMT method exploits rhythm, rhymes, chants, etc., to enrich learning and to increase chances of successful recall.

Musical stimuli improve brain functioning by: providing immediate stimulation and structure to the brain; by introducing timing, grouping, and synchronization for better organization; by recruiting shared or parallel brain systems to assist in performing tasks; by adding emotion and motivation to cognitive processes [11]. Mnemonic strategies, such as visual imagery, chaining, linking pieces of informations in a meaningful order, have been found to be even more effective when applied in conjunction with music rehabilitation techniques. Indeed, melody and rhythm can provide cues to help improve recall capability, yielding better performances than with textual representations.

### 2.4. Musical Attention Control Training

Musical Attention Control Training (MACT) is a music rehabilitation technique for enhancing recovery and improvement of attention faculties. It provides structured active or receptive musical exercises involving precomposed performance or improvisation, in which musical elements cue different musical responses to practice attention functions. When linked with non-musical information, music adds structure and organization, emotion, and appeal to the information in order to increase the probability that attention will be focused, maintained and switched.

Music, as an auditory sensory language, adds a new dimension to the rehabilitation process [12]: by activating right-hemispheric-dominant sustained attention systems, it can have a modulatory influence on spatial attention; it can facilitate divided attention by providing multidimensional stimuli, such as melody and rhythm; it recruits shared or parallel brain systems that assist the frontal lobes with alternating attention; finally, it provides the additional dimensions of emotion and motivation to help facilitate concentration and retain patient focus.

### 2.5. Music in Psychosocial Training and Counseling

Music in Psychosocial Training and Counseling (MPC) is a music rehabilitation technique used for the enhancement of emotional, psychological and social abilities in hospital patients. The MPC technique utilizes musical performance to address issues of affective expression, mood control, cognitive coherence, and appropriate social interaction to facilitate psychosocial functions.

Emotional processing in therapy is needed when emotions, consciously or unconsciously attached to maladaptive behaviours, hinder the development of healthier behaviours, or when emotional experiences such as fear arise and disrupt normal behaviours. Music may help to organize therapeutic experiences around their affective value for the individual, and may lead to processing significant life experiences, managing fears, and setting new goals [13].

Moreover, music is acknowledged to be helpful in the social aspects of rehabilitation [14]. Indeed, the structure of music, since it requires group cooperation, draws people together, and thus yields positive effects on group cohesion, awareness of mood in music, and self-esteem.

Table 1 summarizes the main attributes of the reviewed music rehabilitation techniques.

Note that the mentioned music rehabilitation techniques have also shown to be effective in older patients, in particular for the elderly suffering from Dementia, Parkinson’s Disease, and Alzheimer’s Disease [22,23,24]. Future works shall include such research topics.

## 3. Autonomous Difficulty Adaptation

This section reports the outcome of the academic literature review on AI methods for autonomous game difficulty adaptation, i.e., automatic modification of game features in real time depending on the player’s skills.

The objective is to keep the player engaged throughout the entire game, providing them with an enjoyable yet challenging experience. Indeed, creating an adequate level of challenge is not a trivial task when the game is proposed to players of varying capability. This is a notably important aspect to consider in a clinical setting where the players are children of various ages and bear different pathologies.

### 3.1. Artificial Neural Network

The autonomous difficulty adaptation problem can be tackled by implementing an Artificial Neural Network (ANN), in a Multi Agent System setting [25].

The game adapts according to certain attributes: constant attributes such as age, and dynamic attributes such as number of errors in the game.

A Multi Agent System approach consists in using several intelligent agents connected to each other solving problems that are difficult or impossible for an individual agent [26]. In this application, two agents are used.

The first agent, an ANN, predicts the learner’s performances during a game sequence. The neuron output signal is given by:

$$O=f\left(net\right)=f\left(\sum_{i=1}^{n}w_{i}x_{i}\right),$$

where $W_{i}=\left(w_{1},\cdots ,w_{n}\right)$ is the weight vector and f(net) is the activation function. The variable net is defined as a scalar product of the weight and input vectors: $net=w^{T}x$. Thus, the output value is computed as:

$$O=f\left(net\right)=\left\{\begin{array}{c} 1,\;if\;\;w^{T}>\theta \\ 0,\;otherwise \end{array}\right.\;.$$

where θ is the so called threshold level.

The second agent is the Decision Maker: its role is to make decisions in order to adapt the game according to some parameters, such as the output of the ANN Predictor agent and the number of errors. In particular, such parameters are used to dynamically build a decision tree using the ID3 algorithm [27].

Moreover, the data obtained from experimental sessions can be organized by using an Expectation-Maximization algorithm, in order to cluster the learners’ performances [28].

### 3.2. Probabilistic Approach

The autonomous difficulty adaptation problem can be also tackled using a probabilistic approach [29]. The aim is to maximize the player’s engagement throughout the entire game: their progression is modeled on a probabilistic graph that maximizes engagement as a well-defined objective function. The state for the *k*-th level at the *t*-th trial is denoted as $s_{k,t}$. Three types of transition are possible.

*Level-up transition*: denoting $w_{k,t}$ the win rate and $d_{k,t}^{w}$ the dropout rate after winning, the level-up probability is:

$$Pr\left(s_{k+1,t}|s_{k,t}\right)=w_{k,t}\left(1-d_{k,t}^{w}\right)$$

*Retry transition*: from $s_{k,t}$ the player transits to retrial state $s_{k,t+1}$ only if they win and do not dropout. Denoting $d_{k,t}^{l}$, the dropout rate after losing, the retry probability is:

$$Pr\left(s_{k+1,t}|s_{k,t}\right)=\left(1-w_{k,t}\right)\left(1-d_{k,t}^{l}\right)$$

*Dropout transition*: unless the player makes the above two transitions, they will dropout: thus, the total dropout probability is:

$$Pr\left(dropout|s_{k,t}\right)=w_{k,t}d_{k,t}^{w}+\left(1-w_{k,t}\right)d_{k,t}^{l}$$

Good game design and difficulty adjustment should seek to prevent players falling into the dropout state. The objective is to identify optimal win rates for all states, maximizing the player’s total engagement throughout the entire game. The reward function is the expected total number of rounds that a player will play:

$$R_{k,t}=w_{k,t}\left(1-d_{k,t}^{w}\right)\cdot R_{k+1,t}+\left(1-w_{k,t}\right)\left(1-d_{k,t}^{l}\right)R_{k+1,t}+1$$

An optimization problem finding a set of optimal difficulties over all states is solved:

$$W^{*}=\underset{w}{arg max}R_{1,1}\left(W\right)$$

under the constraint $w_{k,t}\in \left[w_{k,t}^{low},w_{k,t}^{up}\right]$. This problem is solved efficiently with dynamic programming. Denoting the maximum reward $R_{k,t}^{*}$ over all possible difficulty settings, the optimal win rate for state $s_{k,t}$ can be found as:

$$W_{k,t}^{*}=\underset{w_{k,t}}{arg max}w_{k,t}\left(1-d_{k,t}^{w}\right)\cdot R_{k+1,t}^{*}-\left(1-d_{k,t}^{l}\right)\cdot R_{k,t+1}^{*}$$

### 3.3. Dynamic Scripting

Another possible approach to tackle the autonomous difficulty adaptation problem is by using dynamic scripting [30]. This supervised learning approach has been designed for games with several types of opponents, each type with a set of rules manually designed using domain-specific knowledge. When a new opponent is generated, the rules that constitute the script guiding the opponents are taken from the rulebase based on their type. The probability that a rule is selected for a script is proportional to the value of the weight associated with the rule. The rulebase adjusts by amending the values, reflecting the rates of failure or success of the related script rules.

Learning takes place progressively. Upon completion of an encounter, the weights of the rules employed during the encounter are adapted depending on their contribution to the outcome. The rules leading to success have their weights increased, while those leading to failure have their weights decreased. The remaining rules are updated so that the total of all weights in the rulebase remains unchanged. The amount of weight variation ΔW is chosen based on the fitness function that evaluates the performance of a team member as a number in the range [1,0]. The expression of ΔW is given by:

$$\Delta W=\left\{\begin{array}{c} -P_{max}\cdot \frac{b-F}{b}F<b\\ R_{max}\cdot \frac{F-b}{1-b}F\geq b \end{array}\right.\;,$$

where *F* is the fitness, *b* is the break-even value, and $R_{max}$ and $P_{max}$ are the maximum reward and maximum penalty, respectively. Dynamic scripting is used to generate brand new opponent tactics while increasing the level of difficulty of the game’s AI to match the level of experience of the human player.

## 4. Rehabilitative Games

This section introduces the five proposed games, which are based on the music rehabilitation techniques discussed in Section 2.

### 4.1. RhythmRun

The first proposed game, RhythmRun, utilizes Rhythmic Auditory Stimulation as presented in Section 2.1. It is intended for supporting the rehabilitation of children with motor impairments, e.g., paresis which is often caused by stroke, by entraining timing functions and enhancing motor planning and execution since these require spatial and temporal control.

A side-scrolling game is proposed: the player shall avoid some obstacles, whose presence is anticipated by the rhythmic structure of the backing music, i.e., the piece of music played in the background of the game. Thus, the child must understand the temporal pattern of the backing music, in order to predict and dodge incoming obstacles from above or from below.

By touching commands on the screen, the player controls the position of a virtual character who can jump or roll, and whose movement is horizontally constrained and held at a fixed speed.

The action is viewed from a side-view camera angle, and the screen follows the player’s movement up or down. A possible extension is to use a simple MIDI drum-like controller to control the character with their hands and/or feet depending on the specific rehabilitation needs.

The difficulty depends on the speed and complexity of the music piece, and on the number and interchange of the obstacles.

This game trains planning and execution of movements, spatial and temporal control, and the understanding and prediction of rhythm.

### 4.2. SingWings

The second proposed game, SingWings, utilizes Melodic Intonation Therapy as presented in Section 2.2. It is intended for supporting the rehabilitation of children with speech impairments, such as aphasia which is often caused by stroke, and for enhacing neuroplasticity since game engagement requires pitch and spatial control.

A side-scrolling game is proposed: the player shall track a reference melody with their voice. By singing, the child uses their voice to control the position of a virtual character that can move freely in the vertical direction, but is constrained to moving in the horizontal direction at a fixed speed. The control input to the game is the frequency of the note sung by the child, which controls the vertical position of the character. The action is viewed from a side-view camera angle, and the screen follows the player’s movement up or down.

The objective is to avoid certain spatial obstacles that appear on the screen, whose presence obliges the child to sing a higher or lower note, with a certain accuracy, in order to successfully evade the obstacles from above or below.

The difficulty level depends on the speed and harmonic, rhythmic and melodic complexity of the piece of music.

This game trains diction and verbal fluency, pitch control, spatial and temporal control.

### 4.3. MemoryNotes

The third proposed game, MemoryNotes, utilizes Musical Mnemonics Training as presented in Section 2.3. It is intended for supporting the rehabilitation of children with memory impairments, often following stroke and traumatic brain injuries, and for improving memorization in general since the it requires the development of mnemonic techniques.

The player is asked to understand, to interpret, and to remember pairs of images and songs: the objective is to correctly associate each image to the corresponding piece of music. A sequence of pictures appears on the screen, with the name of the associated music. Then each picture is presented again one at a time, and the child attempts to remember and select the linked piece of music. Moreover, it is possible to have the songs repeated by clicking on them.

The difficulty level depends on the number and sorting of the associations, and on whether the associations follow a meaningful reasoning or not.

This game trains memory and provides the opportunity to learn and apply mnemonic strategies.

### 4.4. AwareDrums

The fourth proposed game, AwareDrums, uses Musical Attention Control Training as presented in Section 2.4. It is intended for supporting the rehabilitation of children with attention impairments, often caused by brain tumor and traumatic brain injuries, and for improving attention capabilities in general, which are foundational in good overall cognitive functioning.

In particular, this game focuses on the rehabilitatory potential of rhythm, which enhances timing, organization, and task switching, so that attention can be sustained.

The focus is enacted by using drums virtually through a tablet, and in a possible second phase using real musical instruments, such as simple MIDI percussion instruments.

The player is asked to play drums according to light signals which change in time: depending on the light colour, the child shall play different drums pieces, or stay still. Thus, the child is required to sustain continuous attention, with the aid of music, and to be able to switch between tasks.

The difficulty level depends on the speed and complexity of the piece of music, and on the number of different drum parts to be played.

This game trains attention, fast task-switching, musical rhythm, and capability of establishing and maintaining focus.

### 4.5. MusicEmpathy

The fifth proposed game, MusicEmpathy, utilizes Music in Psychosocial Training and Counseling as presented in Section 2.5. It is intended for the enhancement of empathic, emotional, and social abilities of children. This game combines the development of empathy and the creativity of musical improvisation.

The player selects a character and is then placed in their shoes. The child is then asked to try and understand how the character is feeling, by listening to a piece of music which conveys an emotion such as anger, sadness, excitement, etc. After listening, the child is asked to try play music via an on-screen keyboard expressing the same emotion conveyed by the piece of music, by playing with anger, sadness, excitement, etc. Then, the player is asked to guess which emotion characterizes the piece of music: if the child selects the emotion correctly, an explanation of the reason why the character is feeling the emotion is provided. Then, the child is asked to repeat a short melody extracted by the heard piece of music: if the child plays it back correctly, the next chapter of the character’s storyline is unlocked.

The difficulty level depends on the speed and complexity of the melody extracted from the piece of music to be reproduced.

This game trains emotional self-analysis, creativity, empathy and collaboration with other children.

## 5. Neural Network Classification

This section discusses the Neural Network Classification approach which is proposed in order to automatically adapt the game difficulty parameters of the rehabilitative games presented in Section 4.

The Neural Network (NN) executes a classification in order to select the most suitable difficulty level to apply based on the profile and past performances of the child.

- The input comprises a combination of static attributes, such as age and pathology to recover, and dynamic attributes, such as number of errors and total number of played games.
- The supervised learning is performed in a first phase by deducing the labels from appropriate collaboration with doctors and psychologists.
- The output is the control action selecting the most suitable difficulty level for the next game: the NN shall determine whether to downgrade, to stay in the same level, or to upgrade.

The Griffiths Scales of Mental Development [31] were employed in order to establish the significant parameters to use as inputs of the NN, as shown in Table 2.

The inputs of the Neural Network are defined as in Table 3.

The doctors and psychologists caring for the pediatric patients are provided with reports that are suitably produced by the system in order to evaluate the rehabilitation process. These reports contain the trends of the game attributes shown in Table 1, which describe accordingly the evolution of the child’s performances and cognitive capabilities, with respect to the faculties reported in Table 2. Doctors are also able to validate the actions taken by the system, or to modify them according to the specific current needs of the patient. Moreover, doctors determine how long the games should be played by a child based on the particular necessities of the patient, as well as the maximum allowed game time with respect to safety concerns, such as eye fatigue.

### Synthetic Data Generation

A challenging issue presented by the proposed framework is the small dimension of the training dataset. In the following, a formulation for synthetic data generation via Behavioural Cloning to tackle this issue is presented.

Behavioural Cloning is a sub-class of machine learning concerning how a machine learns to perform a task by attempting to imitate a human performing it [32].

The objective in this study is to describe how Behavioural Cloning can be used to teach a computer to play video games, in order to collect synthetic data [33].

The problem is formulated as a Markov Decision Process:

- It is given a dataset *D* originating from human player games and composed of tuples of the kind (s,a).
- The agent must learn the conditional probability distribution p(a,s), i.e., the probability that a human being performs action *a* when in state *s*.
- After learning this distribution, it can then be used to predict action a∼(a,s) for a given *s*.
- The conditional probability distribution p(a,s) is modeled using a Neural Network which minimizes cross-entropy between predictions and real values of the dataset.
- The state sampling is executed at a constant cadence of 60ms.
- An evalutaion of the results is reported as the percentage of the human being’s score, with 0% corresponding to the minimum of an agent choosing in a random manner, and 100% corresponding to the average human being’s score.

As an explanatory example, the application of the Behavioural Cloning framework to the first game *RhythmRun* is presented:

- Input of the Neural Network:
  s=(DistanceFromObstacle,ObstacleType)
- Output of the Neural Network:
  a=(Idle,Jump,Slide)

## 6. Conclusions

This paper presented a framework for exploiting Artificial Intelligence in pediatric neurorehabilitation through the beneficial effects of music.

Moreover, the outcomes of the reviewed literature were reported regarding music rehabilitation techniques and AI methods for autonomous game difficulty adaptation.

On this basis, five rehabilitative games were designed and introduced based on the selected music rehabilitation techniques. The difficulty settings in the rehabilitation-based games are automatically adapted via implementation of a Neural Network Classification architecture.

Future works will involve the actual testing and validation of the project in a pediatric hospital. In fact, the project has already been presented to the pertinent medical staff of a pediatric hospital, who have expressed strong interest in the topic and availability to test its effectiveness on patients. Moreover, the project has been presented in a public project competition to raise funds for its actual implementation and evaluation.

## Figures and Tables

**Table 1:** Summary of the reviewed music rehabilitation techniques.

	MAIN FEATURES	COGNITIVE FUNCTIONS INVOLVED	ADDRESSED DISEASES	SOME EXPERIMENTAL STUDIES
*RHYTHMIC AUDITORY STIMULATION*	Synchronize movements to auditory stimuli.	Motor Coordination, Rhythm.	Cerebral Palsy, Stroke, Paresis, Multiple Sclerosis.	[3,7,13,15].
*MELODIC INTONATION THERAPY*	Intonate words using a melodic contour.	Diction, Language Processing.	Fluent Aphasia, Non-fluent Aphasia.	[8,13,16,17].
*MUSICAL MNEMONICS TRAINING*	Use music to organize information.	Memory, Attention.	TBI, Stroke, Brain Tumor.	[11,13,18,19].
*MUSICAL ATTENTION CONTROL TRAINING*	Use music to increase focus.	Attention, Rhythm, Task-switching.	Autism, Brain Tumor, Stroke, Dementia.	[12,13,20].
*MUSIC IN PSYCHOSOCIAL TRAINING AND COUNSELING*	Use music for affective expression.	Emotion Processing, Social Skills.	Autism, Depression, Asociality.	[13,21].

**Table 2:** Inputs of the Neural Network.

GAME	GRIFFITHS SCALES	COGNITIVE FACULTIES	NN INPUTS
*All*			Difficulty level of last match, Age.
*RhythmRun*	Hand-eye Coordination, Large Motor Skills.	Motor Coordination, Rhythm.	Hit obstacles, Error mean, Level repetitions.
*SingWings*	Oral-eye Coordination, Communication.	Diction, Language Processing.	Frequency error, Level repetitions, Note duration.
*MemoryNotes*	Learning Fundamentals, Emotionality.	Memory, Attention.	Response time, Number of errors.
*AwareDrums*	Large motor skills, Hand-eye Coordination.	Attention, Rhythm, Task-switching.	Time error, Identification error.
*MusicEmpathy*	Emotionality, Communication.	Emotion processing, Social Skills.	Time error, Note error.

**Table 3:** Definitions of the Neural Network inputs.

INPUT	DEFINITION
Difficulty level of last match	*int*
Age	*int*
Hit obstacles	$\frac{nr\;hit\;obstacles}{nr\;hit\;obstacles}$ ∈[0,1]
Error mean	$\frac{\Sigma |t_{obstacle}-t_{click}|}{nr\;of\;total\;obstacles}$
Level repetitions	*int*
Frequency error	$\frac{\Sigma |f_{objective}-f_{current}|}{nr\;of\;samples}$
Response time	*int*
Number of errors	*int*
Time error	$\frac{\Sigma |t_{beat}-t_{click}|}{nr\;of\;total\;beats}$
Identification error	$\frac{nr\;of\;\mathrm{errors}}{nr\;of\;total\;beats}$ ∈[0,1]
Note error	$\frac{\mathrm{nr}\;\mathrm{of}\;\mathrm{errors}}{nr\;of\;total\;notes}$ ∈[0,1]

## Data Availability

Not applicable.
